# Rose-Mamba-YOLO: an enhanced framework for efficient and accurate greenhouse rose monitoring

**DOI:** 10.3389/fpls.2025.1607582

**Published:** 2025-06-27

**Authors:** Sicheng You, Boheng Li, Yijia Chen, Zhiyan Ren, Yongying Liu, Qingyang Wu, Jianghan Tao, Zhijie Zhang, Chenyu Zhang, Feng Xue, Yulun Chen, Guochen Zhang, Jundong Chen, Jiaqi Wang, Fan Zhao

**Affiliations:** ^1^ Faculty of Data Science, City University of Macau, Macau, Macau SAR, China; ^2^ Department of Applied Informatics, Hosei University, Tokyo, Japan; ^3^ Graduate School of Frontier Sciences, The University of Tokyo, Kashiwa, Japan; ^4^ Department of Environmental Health Sciences, University of California, Los Angeles, Los Angeles, CA, United States; ^5^ Graduate School of Global Environmental Studies, Sophia University, Tokyo, Japan; ^6^ Graduate School of Information, Production and Systems, Waseda University, Kitakyushu, Japan; ^7^ Department of Math and Applied Mathematics, China University of Petroleum-Beijing, Beijing, China; ^8^ Department of Environmental Science, Southwest Forestry University, Kunming, China; ^9^ Data Science and AI Innovation Research Promotion Center, Shiga University, Hikone, Japan

**Keywords:** YOLOv11, mamba, precision agriculture, rose detection, UAV-based monitoring

## Abstract

Accurately detecting roses in UAV-captured greenhouse imagery presents significant challenges due to occlusions, scale variability, and complex environmental conditions. To address these issues, this study introduces ROSE-MAMBA-YOLO, a hybrid detection framework that combines the efficiency of YOLOv11 with Mamba-inspired state-space modeling to enhance feature extraction, multi-scale fusion, and contextual representation. The model achieves a mAP@50 of 87.5%, precision of 90.4%, and recall of 83.1%, surpassing state-of-the-art object detection models. Extensive evaluations validate its robustness against degraded input data and adaptability across diverse datasets. These results demonstrate the applicability of ROSE-MAMBA-YOLO in complex agricultural scenarios. With its lightweight design and real-time capability, the framework provides a scalable and efficient solution for UAV-based rose monitoring, and offers a practical approach for precision floriculture. It sets the stage for integrating advanced detection technologies into real-time crop monitoring systems, advancing intelligent, data-driven agriculture.

## 1 Introduction

The floriculture industry, particularly rose cultivation, plays a vital role in modern agriculture due to its economic and cultural significance (Darras, 2021; Anumala et al., 2021). Accurate monitoring of rose growth stages is essential for optimizing yield, ensuring quality, and responding to fluctuating market demands (Partap et al., 2023). Traditional monitoring methods primarily rely on manual observation, visual inspection, and field surveys, which involve trained horticulturists assessing plant growth, disease symptoms, and blooming stages in person (Mohyuddin et al., 2024). However, traditional methods heavily depend on manual observation, which is labor-intensive, time-consuming, and prone to errors, making them unsuitable for large-scale or high-precision applications (Zhou et al., 2023; Wani et al., 2023). Automated computer vision techniques present a promising alternative to overcome these limitations (Yang and Kim, 2023; Wang and Su, 2022). Among these advancements, deep learning-based object detection models have emerged as the leading tools for automating such monitoring tasks (Li et al., 2024; Lan et al., 2024; Zhou et al., 2023).

Deep learning-based object detection models are the backbone of modern computer vision and can be categorized into single-stage, two-stage, and Transformer-based architectures (Zaidi et al., 2022). Two-stage detectors, such as Faster RCNN and Mask RCNN, deliver high accuracy through a multi-step process of region proposal and refinement (Jiang and Learned-Miller, 2017; He et al., 2017). While effective in complex scenarios, their computational demands and slow inference times make them impractical for real-time applications like UAV-based rose monitoring (Huang et al., 2017; Wu et al., 2019). Transformer-based detectors, including DETR and Swin Transformer, excel at capturing global and long-range dependencies via self-attention mechanisms (Meng et al., 2021; Liu et al., 2021, 2022). However, their high computational complexity and suboptimal performance in small-object detection limit their utility in resource-constrained, agricultural contexts (Khan et al., 2022).

Single-stage detectors, such as YOLO and SSD, offer a more efficient alternative by directly predicting object classes and bounding boxes in one step (Jiang et al., 2022). Among these, the YOLO framework has become a benchmark in real-time object detection due to its remarkable speed, lightweight design, and strong balance between accuracy and efficiency (Diwan et al., 2023). YOLOv10 introduced significant improvements in label assignment and multi-scale detection without using non-maximum suppression (NMS), making it highly suitable for agricultural monitoring with dense object distributions (Alif, 2024). Building upon these advancements, YOLOv11 further enhances feature extraction and inference speed, making it especially suitable for UAV-based agricultural tasks requiring rapid and scalable image processing (Khanam and Hussain, 2024). Despite these advances, even YOLOv11 can face challenges in identifying small or occluded targets such as early-stage rosebuds under complex greenhouse conditions (Liu et al., 2020).

To address these limitations, state-space models (SSMs), such as Mamba (Liu X, et al., 2024; Zhang et al., 2024), provide an efficient solution for modeling long-range dependencies with linear computational complexity (Ma X. et al., 2024). Initially developed for natural language processing, Mamba has shown remarkable versatility across diverse domains by efficiently modeling sequential and contextual relationships (Gu and Dao, 2023; Qu H, et al., 2024). Recent studies show that integrating Mamba into object detection frameworks leads to enhanced robustness, particularly in detecting small and occluded objects (Wang et al., 2024).

This study introduces a novel hybrid model combining Mamba and YOLOv11 to tackle key challenges in rose detection across different growth stages. Mamba’s ability to model long-range dependencies complements YOLOv11’s efficiency and real-time capabilities. The proposed approach integrates Mamba-inspired modules to improve feature extraction, multi-scale fusion, and contextual understanding, providing a robust and computationally efficient solution for UAV-based rose monitoring (Chen et al., 2025). This integration effectively tackles challenges such as scale variability and complex backgrounds, advancing the accuracy and reliability of rose detection (Zhao et al., 2025).

Our contributions are as follows:

1. Integration of Mamba into YOLO: We combine Mamba-inspired state-space modeling with YOLO’s real-time detection framework to achieve enhanced feature extraction and computational efficiency.
2. Optimized detection for floriculture: The proposed hybrid model addresses challenges such as occlusions and small-object detection, making it highly suitable for rose detection across different growth stages.
3. Comprehensive validation: Extensive experiments demonstrate the model’s robustness under degraded input conditions and its scalability across different datasets, showcasing its practical utility.
4. Practical applicability: The model balances detection precision, recall, and computational efficiency, making it scalable for real-world applications in floriculture.

## 2 Related work

### 2.1 Flower detection

Deep learning-based approaches have been widely adopted for automated flower detection across various agricultural applications. Shang et al. (2023) introduced a lightweight YOLOv5s-based model for detecting apple flowers in natural environments. By integrating ShuffleNetv2 into the backbone and a Ghost module in the neck, the model effectively reduced computational complexity while maintaining accuracy. With a precision of 88.4% and recall of 86.1%, it proved highly efficient for real-time applications such as robotic flower thinning. Rahim et al. (2022) developed a segmentation-based method for grapevine flower quantification using Mask R-CNN. Their two-step approach first localized inflorescences and then detected individual flowers, achieving F1 scores of 0.943 and 0.903, respectively. This framework demonstrated high accuracy in yield estimation, showcasing the potential of deep learning for vineyard monitoring.

Beyond agricultural applications, Shirai et al. (2022) applied UAV-based detection techniques to monitor Convallaria keiskei colonies, an endangered plant species. Their model combined Convolutional Neural Networks (CNNs) with fuzzy c-means clustering to enhance classification accuracy, improving the F-measure by 22.0% over conventional CNN approaches. Similarly, Petrich et al. (2020) developed a CNN-based detection method for Colchicum autumnale, a toxic flowering plant found in pastures. Through data augmentation, their model achieved an 88.6% detection rate, demonstrating the effectiveness of deep learning for large-scale vegetation monitoring.

While these studies demonstrate significant progress in flower detection, several challenges remain. Real-time detection models offer efficiency and speed but often face difficulties in handling occlusions and intricate floral structures (Malakar et al., 2023; Ma Y. et al., 2024). UAV-based detection techniques enhance coverage and automation but are influenced by image resolution, environmental variability, and processing constraints (Zhou et al., 2021; Zhao et al., 2024a). Overcoming these limitations is essential to improve detection accuracy, adaptability, and efficiency in floriculture applications.

### 2.2 State space models

State Space Models (SSMs) have long been employed to describe dynamic systems in fields such as control theory, signal processing, and economics (Zhou et al., 2023). More recently, they have emerged as a powerful framework in deep learning, particularly for sequence modeling tasks, including time series forecasting, natural language processing (NLP), and video understanding (Gu et al., 2022). Unlike traditional recurrent architectures, which suffer from vanishing gradients and inefficient memory usage, SSMs provide an effective mechanism for capturing long-range dependencies while maintaining linear computational complexity, making them highly scalable for large-scale applications (Gu et al., 2021).

A breakthrough in deep learning came with the introduction of Structured State Space Sequence Models (S4) by Gu et al. (2021) S4 demonstrated the ability of SSMs to efficiently model long-range dependencies while scaling effectively with sequence length. It introduced parameterized state-space layers that enhanced sequence modeling, laying the foundation for further advancements. Building on this, Smith et al. (2022) developed S5, which incorporated multi-input multi-output (MIMO) SSMs and an efficient parallel scan mechanism to further improve training and inference efficiency. These innovations positioned SSMs as a compelling alternative to traditional deep learning architectures, particularly for tasks requiring efficient long-sequence modeling.

Mamba, introduced by Gu and Dao (2023), represents the latest advancement in SSMs, extending the principles of S4 and S5. By integrating a selective state-space mechanism with time-varying parameters, Mamba enhances sequence modeling without the quadratic complexity of Transformers (Qu S, et al., 2024). It achieves comparable or superior performance to Transformer models in NLP tasks while maintaining linear complexity, making it highly effective for large-scale sequential processing (Patro and Agneeswaran, 2024). Mamba’s hardware-aware optimization further boosts its efficiency, enabling real-world applications that require both speed and scalability.

Encouraged by Mamba’s success in NLP, researchers have expanded its application to computer vision. Vision Mamba, an early attempt to develop a structured state-space model as a visual backbone, adapts Mamba’s sequential modeling capabilities for image-based tasks. It incorporates bidirectional scanning mechanisms to handle spatial dependencies in images, enabling effective feature representation while maintaining computational efficiency (Rahman et al., 2024). Liu et al. (2024) further refined this approach with VMamba, which integrates 2D-Selective-Scan (SS2D) to enhance spatial relationship modeling. These advancements demonstrate the potential of SSM-based architectures in computer vision, proving that Mamba-inspired models can efficiently process large-scale visual data while retaining the scalability and efficiency advantages inherent to state-space models.

Initially, research on SSMs in vision tasks was primarily focused on image classification and segmentation (Liu X, et al., 2024; Zhao et al., 2024b). However, recent studies have extended their applications to more complex domains such as remote sensing and real-time object detection (Ma B, et al., 2024; Wang et al., 2024; Zhao et al., 2024c). Mamba-based architecture has been proven beneficial in UAV-based monitoring and agricultural applications, where high-resolution image sequences require efficient processing without excessive computational overhead. The ability of SSMs to capture long-range dependencies while maintaining scalability makes them an attractive alternative to CNNs and Transformers, especially in resource-constrained environments (Qu H, et al., 2024).

### 2.3 Real-time object detectors

Real-time object detection has become a cornerstone of modern computer vision, enabling rapid and precise recognition of objects in dynamic environments (Rane, 2023). Traditional object detection techniques, such as Haar cascades, Histogram of Oriented Gradients (HOG), and Deformable Part Models (DPM), are limited by high computational costs and poor adaptability (Xu et al., 2014; Hosain et al., 2024). The emergence of deep learning, such as CNNs, revolutionized object detection by introducing robust feature extraction and hierarchical representation learning, significantly enhancing performance and efficiency (Aziz et al., 2020).

Among deep learning-based approaches, You Only Look Once series (YOLO) has played a pivotal role in transforming real-time detection with its end-to-end processing pipeline (Liang et al., 2022). Unlike earlier region-based methods, YOLO performs direct regression for object localization and classification in a single forward pass, enabling significant improvements in speed while maintaining competitive accuracy (Sirisha et al., 2023). Over multiple iterations, the YOLO framework has undergone substantial refinements to balance detection performance and computational efficiency.

YOLOv1 introduced single-pass detection but struggled with small-object recognition (Hussain, 2024). Subsequent versions introduced enhancements gradually: YOLOv2 incorporated anchor boxes for improved localization; YOLOv3 adopted multi-scale feature maps; and YOLOv4 refined training strategies with optimizations such as CSPDarknet-53 and self-adversarial training (Alif, 2024). Later iterations, including YOLOv5, YOLOv6, and YOLOv7, focused on model scaling, re-parameterization techniques, and decoupled head structures to enhance computational efficiency (Ali and Zhang, 2024; Li J, et al., 2025). YOLOv8 further advanced feature aggregation and bounding box regression, maintaining strong real-time performance across diverse applications (Sohan et al., 2024; Zhao et al., 2024d). YOLOv9 adjusted receptive fields to improve multi-scale detection, while YOLOv10 incorporated an NMS-free training approach with dual label assignments, enhancing both accuracy and inference speed (Yaseen, 2024; Wang et al., 2024).

The latest iteration, YOLOv11, introduces several key architectural advancements, including the C3k2 block, Spatial Pyramid Pooling - Fast (SPPF), and the Convolutional block with Parallel Spatial Attention (C2PSA). These enhancements collectively improve feature extraction, multi-scale processing, and computational efficiency (Khanam and Hussain, 2024). YOLOv11 optimizes parameter efficiency while maintaining a strong balance between accuracy and speed, making it adaptable for deployment across various computational environments, from edge devices to high-performance computing platforms. By refining its architecture, YOLOv11 further advances real-time object detection, offering a highly scalable and precise solution for high-speed vision applications (Jegham et al., 2024).

The continual advancement of real-time object detectors underscores the need for models that strike an optimal balance between speed, accuracy, and computational efficiency. As computer vision applications expand across industries such as autonomous vehicles, surveillance, and precision agriculture, the development of robust and adaptable detection frameworks remains a critical focus in AI research (Janai et al., 2020; Tian et al., 2020).

## 3 Materials and methods

To address the challenges of rose detection across different growth stages, this study integrates advanced modules and frameworks into a unified model and evaluates its performance systematically. This chapter outlines the datasets, model architecture, training methodologies, and evaluation metrics employed to develop and validate the proposed approach.

### 3.1 Dataset

This study utilized the *RoseBlooming* dataset, specifically designed for stage-specific rose detection and tracking in greenhouse environments (Shinoda et al., 2023). The dataset features high-resolution annotated images of two rose varieties, Rosa hybrida hort. ‘Samourai 08’ and ‘Blossom Pink,’ cultivated under controlled conditions at the Kizu Experimental Farm of Kyoto University. With comprehensive annotations of roses at different growth stages, it serves as a valuable resource for evaluating object detection models.

**Figure 1:**
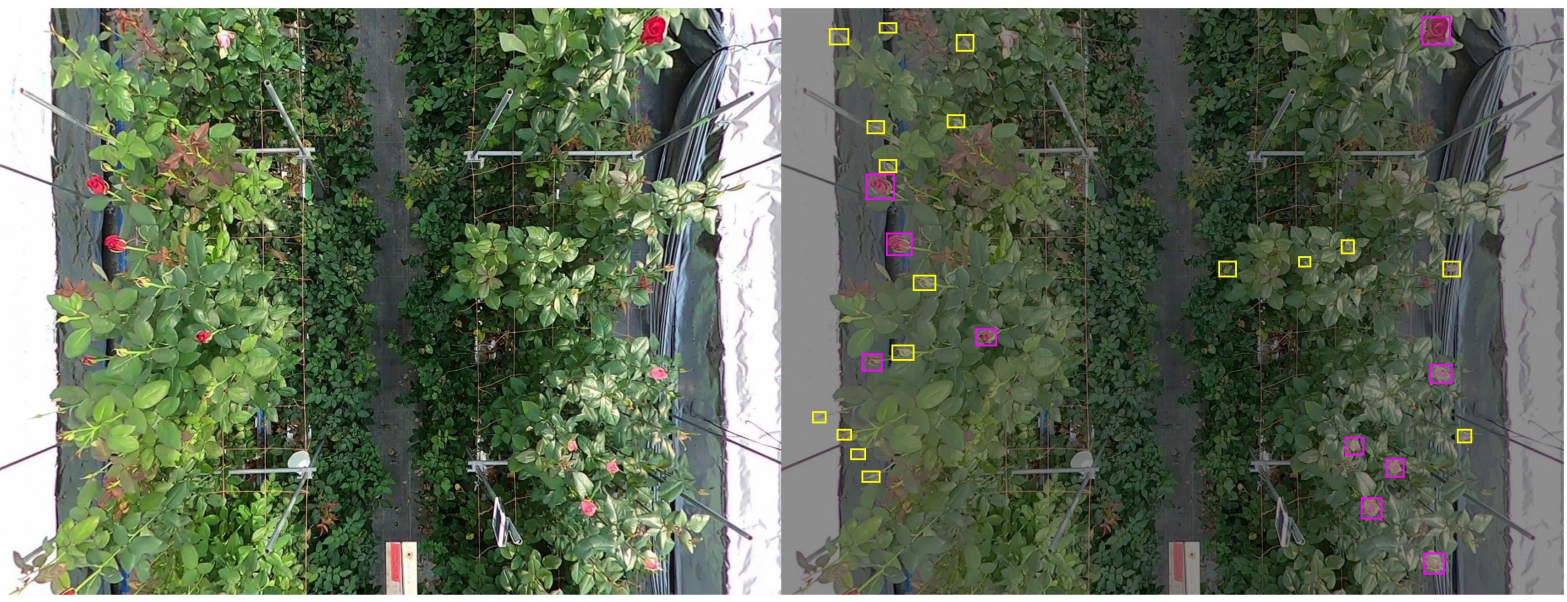
Example image from the RoseBlooming dataset (left), and annotated version from the labeling interface (right).

The dataset categorizes roses into two growth stages: rose_small and rose_large. The rose_small category encompasses roses from the bud stage to the point where petals remain aligned with the flower’s central axis, while the rose_large category includes fully bloomed roses with petals extending visibly beyond this alignment. Annotations were generated using Microsoft’s VOTT tool to ensure consistent bounding box labels for each growth stage. **Figure 1** illustrates annotated examples, where pink bounding boxes represent rose_large and yellow bounding boxes denote rose_small.

**Figure 2:**
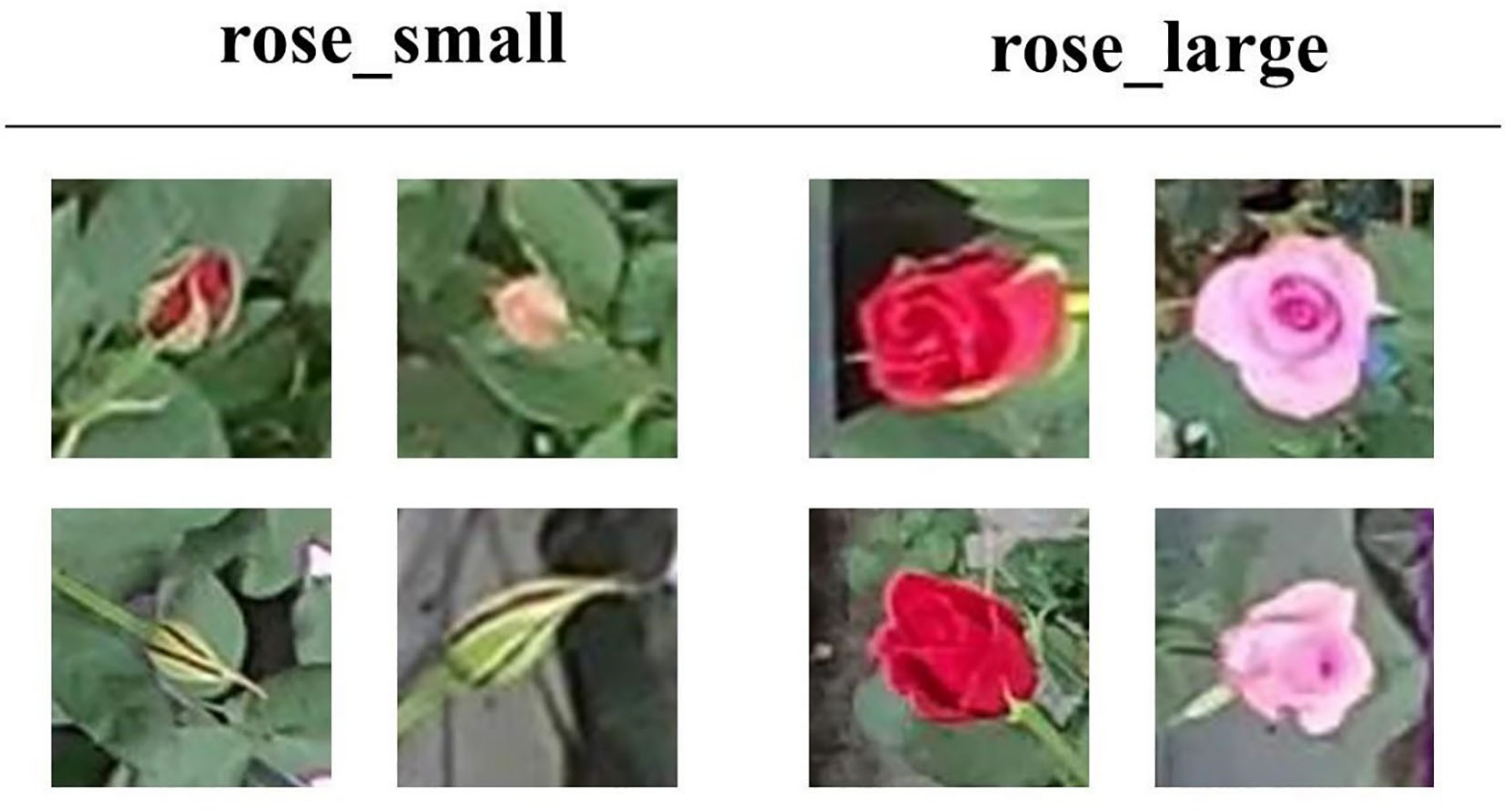
Sample images from the dataset, showcasing the two annotated growth stages.

As shown in **Figure 2**, the dataset contains representative image samples of roses at different developmental stages, clearly distinguishing between *rose_small* and *rose_large* categories based on petal structure and growth characteristics.

The dataset consists of 519 images, which are divided into training, validation, and test sets in a 6:2:2 ratio. This structured division provides sufficient data for both model training and evaluation. With over 7,000 annotated bounding boxes, the dataset captures the density and variability of roses in realistic greenhouse environments, covering a range of developmental stages and environmental conditions.

### 3.2 Model architecture

Object detection methods have seen significant advancements, with single-stage approaches like YOLO gaining prominence for their efficiency in real-time applications (Alif and Hussain, 2024). Unlike two-stage methods, which rely on region proposals followed by refinement, single-stage frameworks directly predict object locations and classes in one pass (Zhang et al., 2018; Meng et al., 2021). This design improves computational efficiency, making it well-suited for high-speed and scalable tasks.

Expanding upon previous advancements, YOLOv11 integrates novel architectural components, including the C3k2 block, SPPF, and C2PSA, further refining feature extraction and computational efficiency. These enhancements enable YOLOv11 to achieve state-of-the-art performance in feature extraction, multi-scale processing, and computational efficiency (Alif, 2024; Jegham et al., 2024).

Despite these advancements, applying YOLOv11 to UAV-collected rose imagery presents challenges due to complex backgrounds, high dynamic ranges, and densely packed objects (Tang et al., 2023). These issues result in difficulties such as occlusion handling, accurate small-object detection, and effective feature fusion across scales. To address these limitations, this study introduces Rose-Mamba-YOLO, a hybrid architecture built upon YOLOv11 with the following four key enhancements: 1). Integration of Mamba-based modules to efficiently capture long-range dependencies. 2). Enhanced spatial attention mechanisms to handle densely distributed roses and mitigate occlusions. 3). Improved multi-scale feature fusion to address scale variations in rose detection. 4). Contextual feature integration to improve the representation of small objects like rose buds.

**Figure 3:**
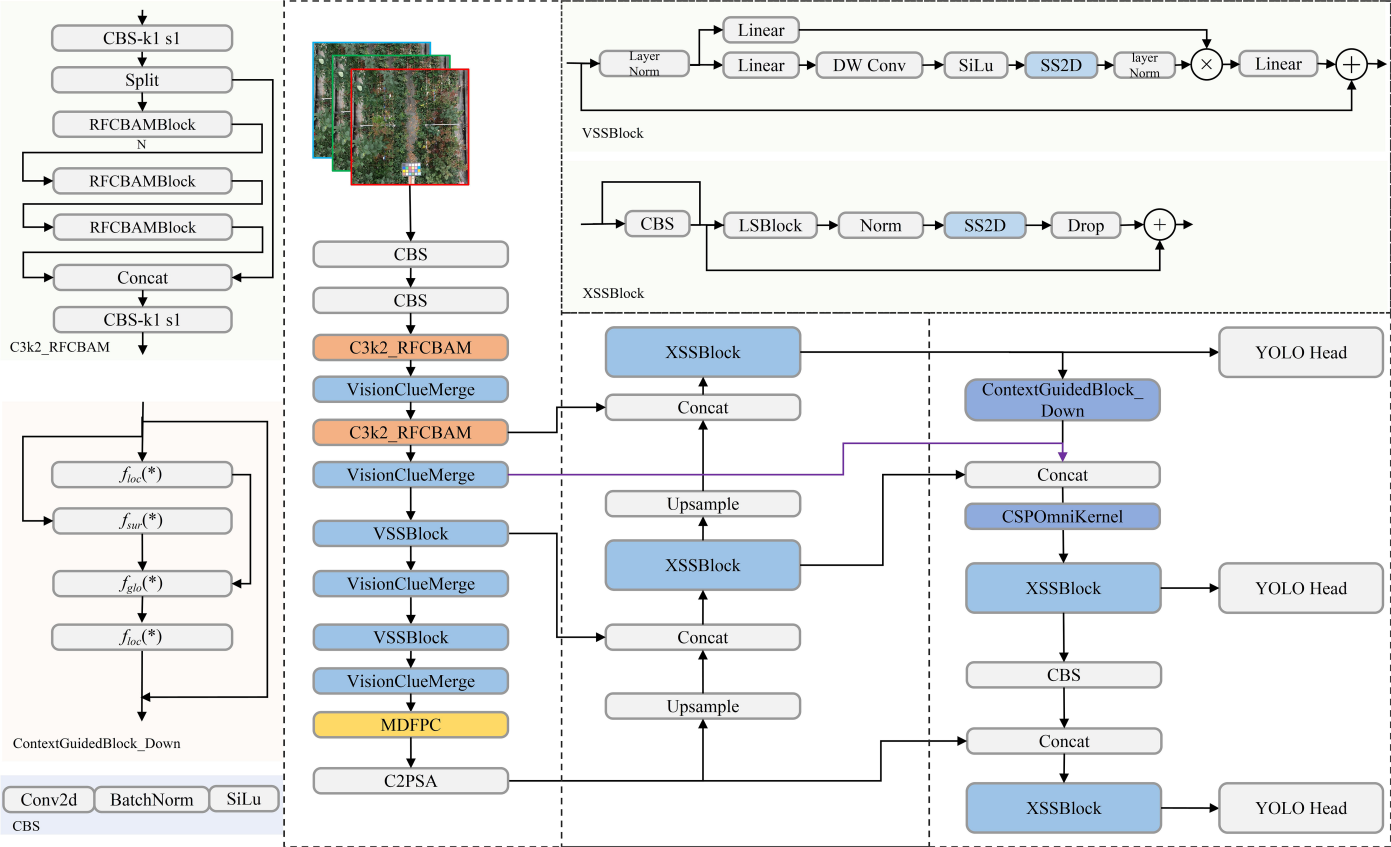
Schematic Representation of Rose-Mamba-YOLO Architecture. Components adapted from YOLOv11 and modules inspired by the Mamba model are labeled by name and function, with their origins further elaborated in this Section.

These enhancements collectively improve YOLOv11’s robustness and accuracy, making Rose-Mamba-YOLO well-suited to the challenges of stage-specific rose detection in greenhouse environments. A schematic representation of the proposed model is shown in **Figure 3**, illustrating the integration of Mamba-based modules and the architectural advancements over standard YOLOv11. The following sections provide detailed explanations of each innovation and its contributions to the architecture and performance of Rose-Mamba-YOLO.

#### 3.2.1 Mamba-based modules

**Figure 4:**
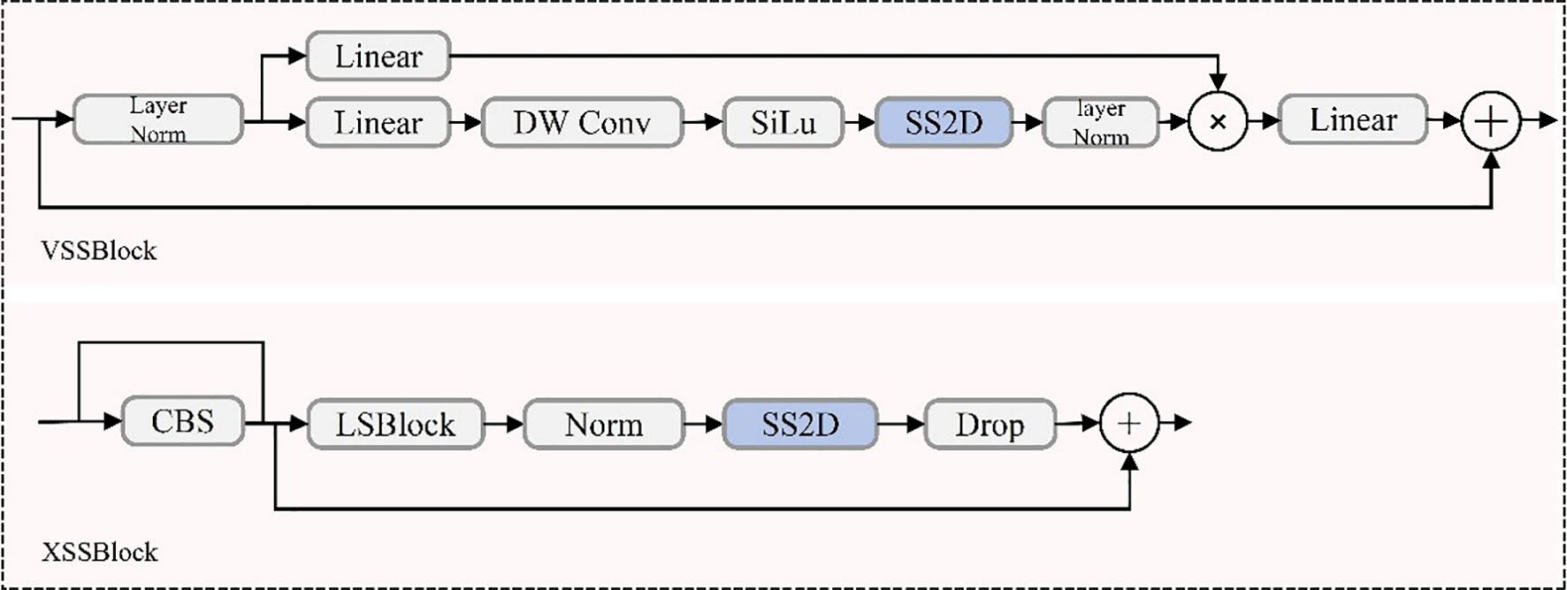
VSSBlock and XSSBlock structure.

The integration of Mamba-based modules into the YOLOv11 backbone significantly enhances detection capabilities for UAV-captured rose images. In particular, the original C3k2 backbone of YOLOv11 was replaced by the VSSBlock and VisionClueMerge components derived from the Mamba model, enabling the redesigned backbone to leverage state-space modeling while significantly improving feature extraction and multi-scale representation (Khanam and Hussain, 2024; Wang et al., 2024). Among these, the VSSBlock serves as a core module, leveraging an optimized State Space Model (SSM) and depthwise separable convolution techniques (Ma X, et al., 2024). This design enables the effective extraction of complex features, such as object shapes, textures, and spatial relationships, addressing challenges like occlusions and densely packed arrangements (Feng et al., 2024). The VSSBlock’s structure, shown in **Figure 4**, highlights its integration of state-space modeling and convolutional operations, contributing to enhanced feature extraction.

To further improve multi-scale feature processing, the XSSBlock is integrated into the neck of the YOLOv11 architecture. This module is especially effective in detecting small objects, such as rose buds, which frequently appear in low-resolution regions of UAV imagery. By incorporating pyramid attention mechanisms and a Feature Pyramid Network (FPN) configuration, the XSSBlock refines multi-scale features, enabling accurate detection of both large-scale, high-resolution targets and small-scale, low-resolution objects (Zhu et al., 2022). As depicted in **Figure 4**, the XSSBlock combines multi-scale attention with feature refinement, ensuring robust feature representation across varying resolutions and scales.

The combined integration of the VSSBlock, XSSBlock, and VisionClueMerge significantly enhances YOLOv11’s feature extraction and multi-scale processing while maintaining computational efficiency and real-time applicability. These modules were not appended as auxiliary components but were directly embedded and substituted into the core architecture, ensuring seamless integration of Mamba principles within the YOLOv11 framework.

This adaptation improves the model’s robustness and stability, particularly for UAV-based rose detection tasks that require precision and efficiency in dynamic environments. By replacing the standard C3k2 backbone with Mamba-based structures, the proposed model achieves substantial improvements in detection accuracy, computational performance, and scalability (He et al., 2025).

#### 3.2.2 Spatial attention mechanisms

**Figure 5:**
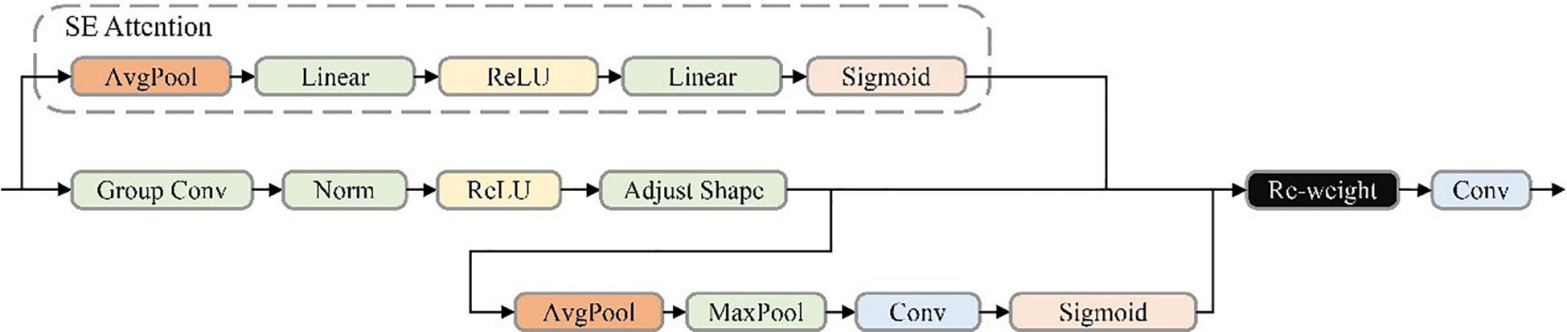
RFCBAMBlock model structure diagram.

The Receptive Field Block (RFB), inspired by the human visual system’s receptive field mechanisms, enhances a network’s ability to process multi-scale features effectively (Zhang M, et al., 2023). Building on this concept, a novel module named RFCBAMBlock is proposed, as illustrated in **Figure 5**, which incorporates spatial attention into receptive field modeling. This newly designed structure enables the network to dynamically reweight features across multiple receptive field regions—an integration not previously explored in YOLO-based detectors, to the best of our knowledge. By addressing the parameter-sharing limitations associated with varying convolutional kernel sizes, RFCBAMBlock demonstrates strong performance in dense-object recognition tasks, particularly within structured agricultural imaging environments.

**Figure 6:**
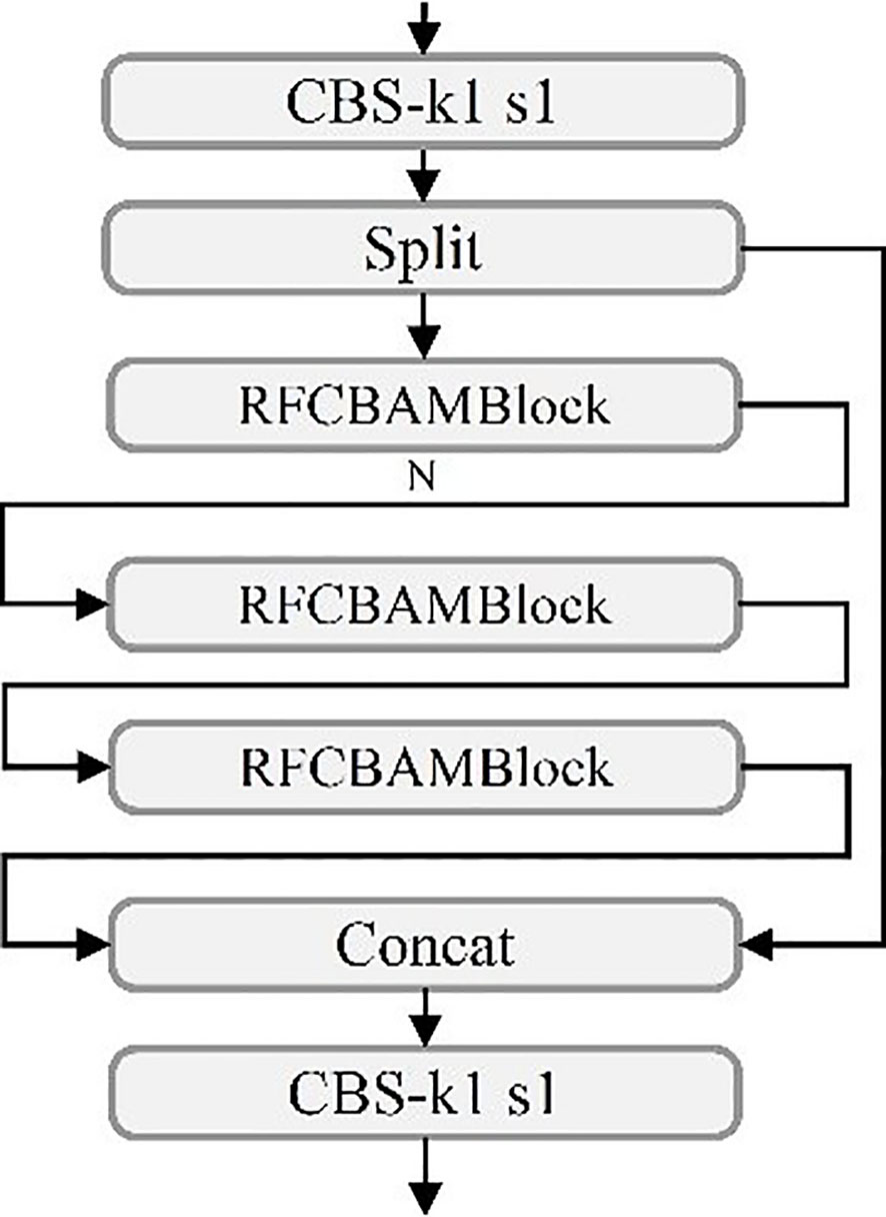
C3k2_RFCBAM model structure diagram.

In the YOLOv11 architecture, the existing C3k2 module exhibits limitations in feature extraction, particularly for detecting small objects in UAV-based rose imagery (Zhang et al., 2025). To address this issue, the RFCBAMBlock is incorporated into the C3k2 module, resulting in the improved C3k2_RFCBAM module (**Figure 6**). By leveraging spatial attention mechanisms, the C3k2_RFCBAM module adaptively adjusts the receptive field size, significantly enhancing the network’s capability to process multi-scale features.

From a feature extraction perspective, the inclusion of RFCBAMBlock greatly improves C3k2’s ability to capture multi-scale features. Traditional convolutional layers, constrained by fixed receptive fields, struggle to effectively handle features at varying scales, particularly for small objects (Ma J, et al., 2024). The RFCBAMBlock resolves this limitation by allowing flexible adjustments to the receptive field size, enabling the C3k2_RFCBAM module to efficiently handle diverse shapes and scale variations of small objects, which are critical in UAV-based rose detection tasks.

Additionally, the integration of spatial attention mechanisms within the RFCBAMBlock enhances feature extraction by dynamically prioritizing convolutional kernel responses across different receptive field regions. This design mitigates parameter-sharing issues caused by varying kernel sizes, enabling more precise feature extraction in complex scenes. By replacing the original C3k2 module with the enhanced C3k2_RFCBAM, YOLOv11 achieves significant improvements in feature extraction, multi-scale processing, and small-object detection capabilities, demonstrating superior performance in challenging environments.

#### 3.2.3 Multi-scale feature fusion

**Figure 7:**
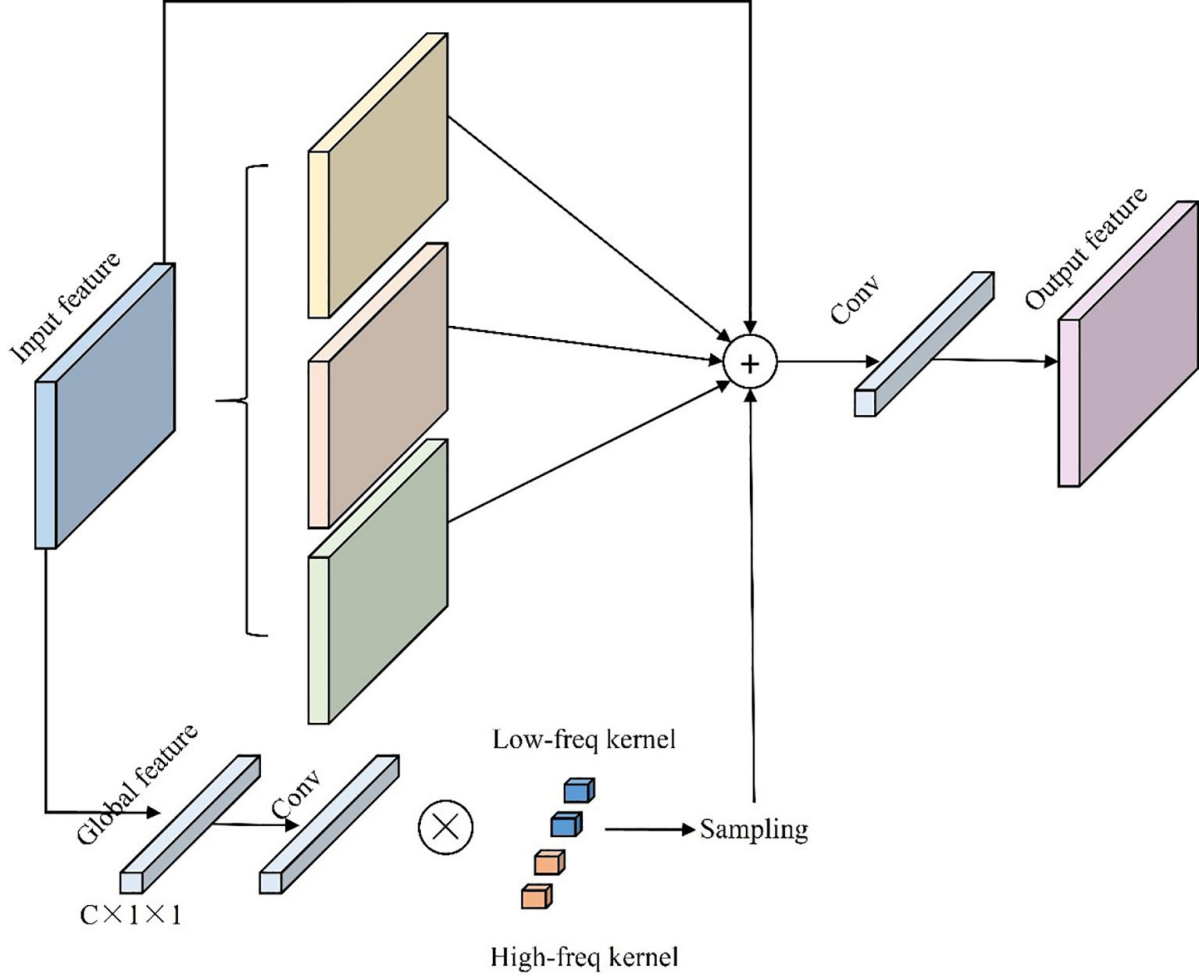
MDFPC model structure diagram.

In UAV-based detection tasks, Spatial Pyramid Pooling - Fast (SPPF) is commonly used to accelerate pooling computations and facilitate multi-scale feature fusion (Lu and Sun, 2025). However, pooling operations often result in the loss of fine-grained details, particularly when addressing extreme scale variations, which can compromise detection accuracy (Chen et al., 2024). To overcome these limitations, the Multiscale Dilated Feature Pyramid Convolution (MDFPC) module is proposed, as shown in **Figure 7**. MDFPC utilizes dilated convolutions with varying dilation rates (6, 12, 18) to enhance global context awareness while preserving fine-grained details, demonstrating significant advantages in small-object detection (Zhao et al., 2019).

The core innovation of the MDFPC module lies in its use of dilated convolutions to expand the receptive field without increasing computational overhead. By introducing “holes” within the convolutional kernels, dilated convolutions effectively enlarge the receptive field while maintaining computational efficiency. Convolutions with lower dilation rates focus on extracting local features from small objects, capturing fine details critical for detecting rose buds, while higher dilation rates expand the receptive field to improve global context understanding for larger objects and background features. This dual capability enables MDFPC to address the scale variability challenges inherent in UAV imagery of roses.

The module begins with a 1×1 convolutional layer to compress input feature channels, reducing computational complexity while preserving essential information. The compressed features are then processed through dilated convolutions with varying dilation rates, allowing the extraction of multi-scale information. These multi-scale features are subsequently concatenated and fused using another 1×1 convolutional layer, integrating extracted features while further compressing dimensions to balance computational efficiency and model performance. Unlike traditional pooling operations that often lead to fine-grained detail loss, MDFPC retains critical information, making it particularly effective for small-object detection (Gholamalinezhad and Khosravi, 2020).

By expanding the receptive field without increasing computational overhead, MDFPC captures a broader range of contextual information while maintaining efficiency. Although compressing feature dimensions can result in minor information loss, especially for small-object features, extensive experimentation has fine-tuned the compression ratio to achieve an optimal balance between efficiency and detection accuracy. This enhancement is particularly beneficial for UAV-based rose detection, where small objects like rose buds coexist with larger-scale features, requiring precise multi-scale processing to ensure accurate detection across varying resolutions.

#### 3.2.4 Contextual feature integration

**Figure 8:**
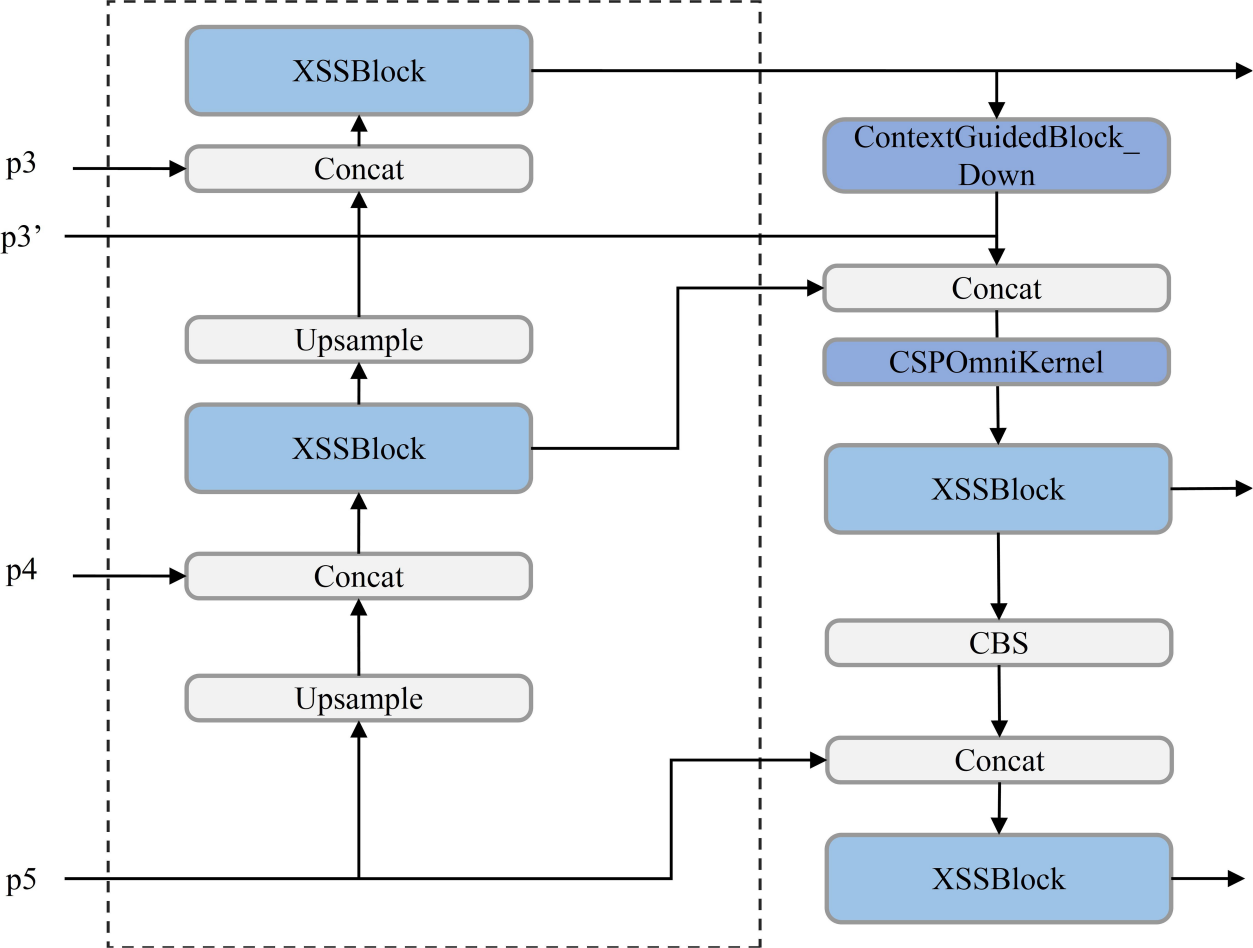
CMFPN model structure diagram.

UAV-based rose detection tasks present significant challenges, particularly in capturing fine-grained features at the P3, P4, and P5 layers of the Feature Pyramid Network (FPN) (Lin et al., 2017). These layers often struggle to extract the detailed information required for small-object detection. Traditional solutions attempt to mitigate this issue by introducing an additional P2 layer before the P3 layer to enhance small-object detection capabilities (Oksuz et al., 2020). However, such approaches often come at the cost of increased computational demands and extended post-processing times, reducing overall efficiency. To address these limitations, the Contextual Multi-Scale Feature Pyramid Network (CMFPN) is proposed, as shown in **Figure 8** (Li et al., 2022).

**Figure 9:**
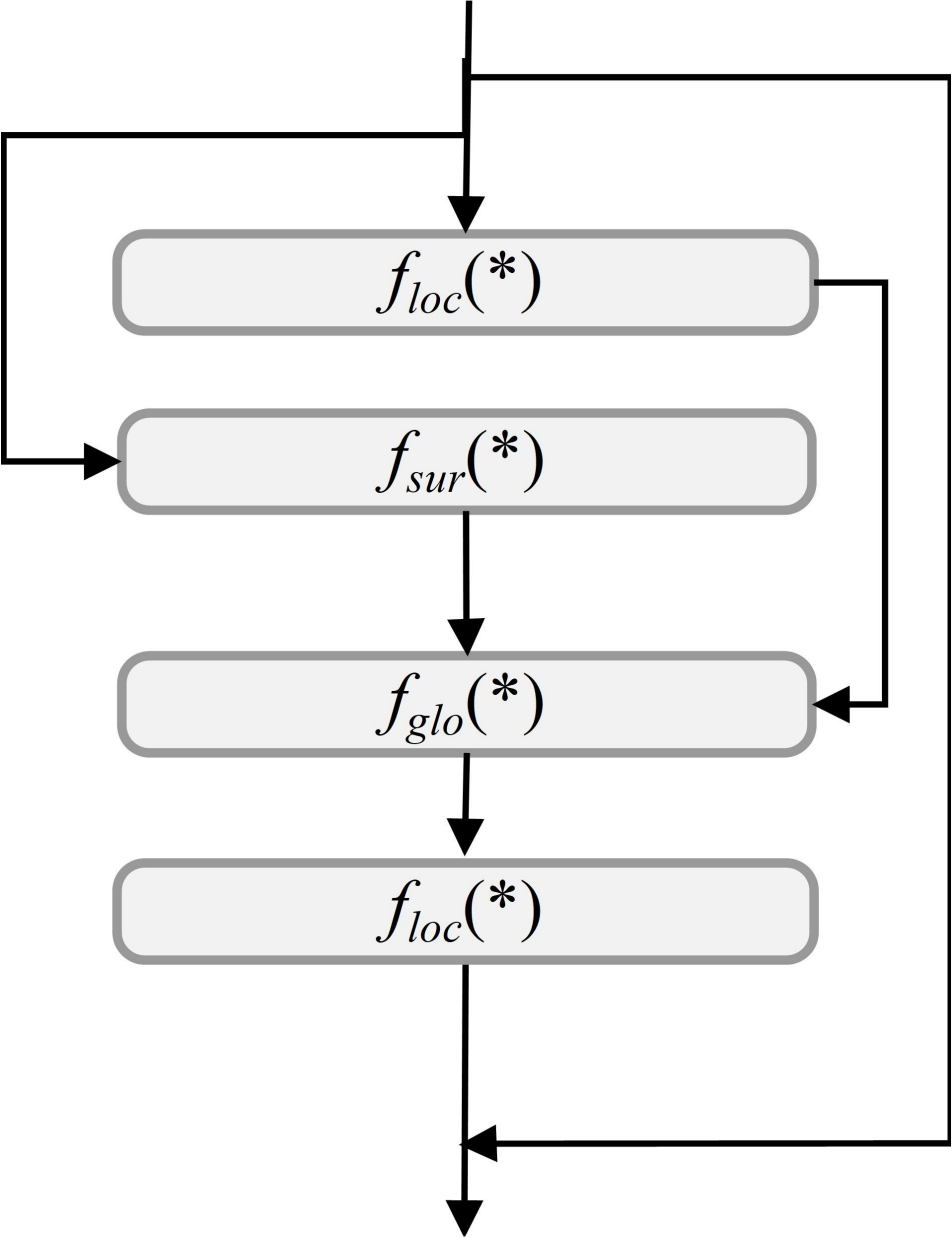
CGBD model structure diagram.

The CMFPN enhances the PAN-FPN structure by introducing a new feature map, P3’, at the P3 and P4 layers, specifically designed to improve the detection of small objects, such as rose buds. The P3’ feature map is processed through the ContextGuidedBlock_Down (CGBD) module, which strengthens contextual awareness for small objects in UAV-captured rose images (Wu et al., 2020). The structure of the CGBD module is illustrated in **Figure 9**. By integrating contextual information, the P3’ feature map captures enriched features for small objects, which are then fused with the upsampling layer (Upsample) and the original P3 layer features. These fused features are passed into the CSPOmniKernel module for multi-scale feature fusion.

**Figure 10:**
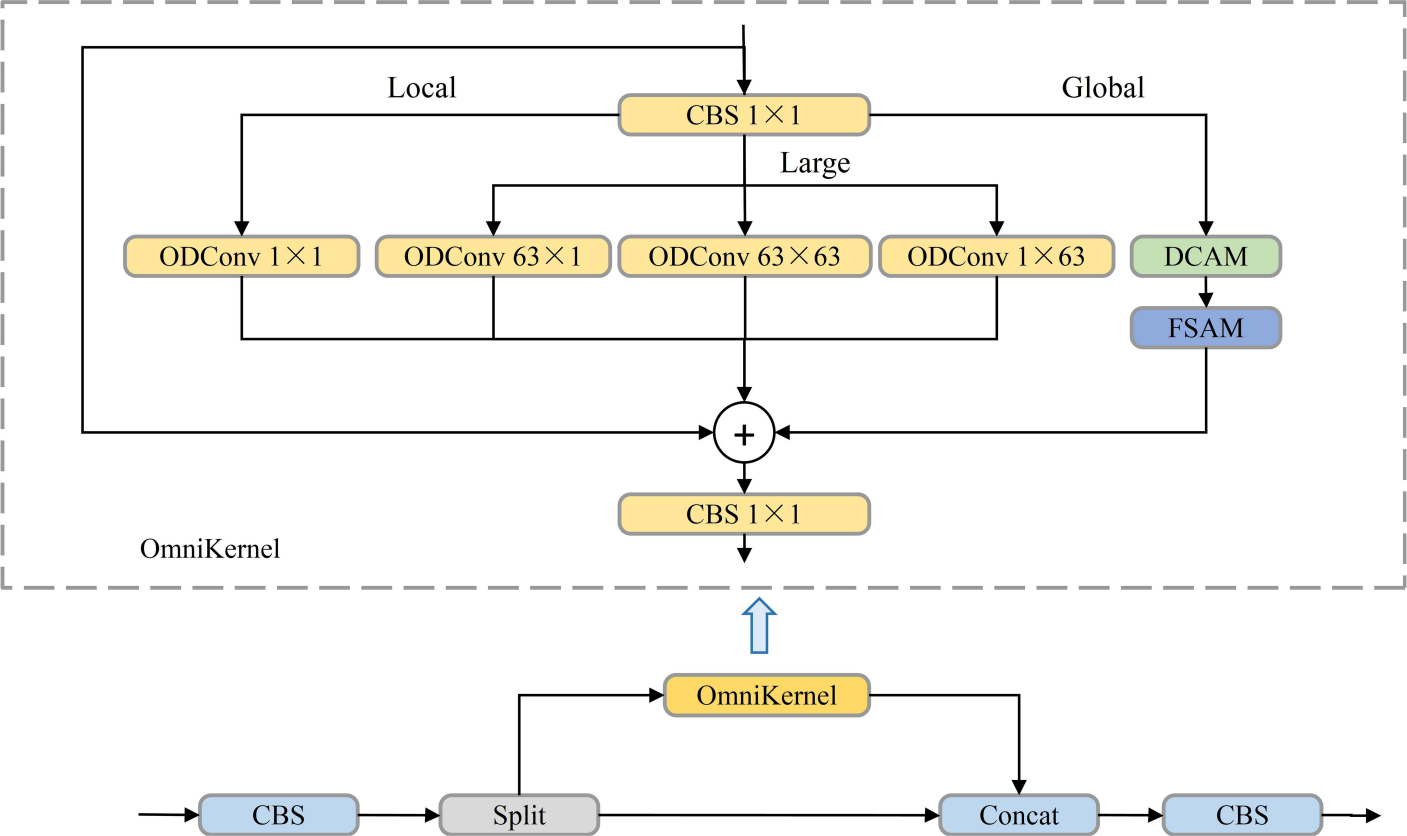
CSPOmniKernel model structure diagram.

The CSPOmniKernel module, depicted in **Figure 10** combines the OmniKernel algorithm with the Cross Stage Partial (CSP) concept to enhance feature expression, improve gradient flow, and reduce model complexity (Cui et al., 2024). The CSP design facilitates cross-stage feature fusion, alleviating gradient vanishing issues while lowering computational complexity and reducing the parameter count. Meanwhile, the OmniKernel algorithm dynamically adjusts kernel sizes to expand the receptive field, allowing the model to better handle variations between small objects and large-scale targets. The CSPOmniKernel module integrates global, large-scale, and local branches, fusing them through addition, followed by a 1×1 convolution to effectively combine multi-scale features. This design enables simultaneous processing of features across scales, significantly improving small-object detection precision.

The introduction of the CMFPN structure enables superior small-object detection without compromising computational efficiency. By combining P3’ features, the CGBD module for contextual enhancement, and the CSPOmniKernel module for multi-scale fusion, the model captures detailed and diverse features across scales.

#### 3.3 Network training and optimization

The experimental environment and configurations used in this study are summarized in **Table 1**. The experiments were conducted on an Ubuntu 18.04 operating system with 64GB of memory, utilizing an NVIDIA GeForce RTX 4090 GPU with 24GB of memory. The CPU used was an Intel(R) Xeon(R) Platinum i9-13900k processor. Model training was performed using the PyTorch 1.10 deep learning framework, with GPU acceleration provided by CUDA 11.1 and CUDNN 8.0.4.

**Table 1:** Experimental environment parameters.

Parameter	Value
Operating system	Ubuntu 18.04
System architecture	32-bit
RAM	64GB
GPU	GeForce RTX 4090
GPU memory	24GB
CPU	Intel(R) Xeon(R) Platinum i9-13900k
Deep learning framework	PyTorch 1.10
CUDA version	11.1
CUDNN version	8.0.4

The input image size for model training was set to 640×640 pixels, with an initial learning rate of 0.01. The Stochastic Gradient Descent (SGD) optimizer was employed, with a momentum of 0.937 and a weight decay of 0.0005. To enrich the diversity of detection backgrounds and enhance robustness, Mosaic data augmentation was applied during training. This process involved randomly selecting, flipping, and scaling four images, which were then stitched together into a composite image. These augmentation strategies effectively simulate real-world variability such as changes in lighting, object occlusion, and background complexity, contributing to the model’s robustness in practical deployment scenarios.

To further mitigate overfitting and improve generalization, label smoothing was applied with a smoothing factor of 0.01. The model was trained for 200 epochs with a batch size of 8, while 8 worker threads were utilized to optimize data loading performance. These training configurations, combined with the proposed architecture, ensured efficient learning and convergence while maintaining robust generalization to the complex and diverse UAV-captured rose images.

### 3.4 Evaluation metrics for object detection

Evaluating the performance of detection model involves analyzing multiple metrics that measure both prediction accuracy and localization precision (Qu S, et al., 2024). Among these, precision and recall serve as foundational indicators of model performance. Precision quantifies the proportion of correctly predicted positive instances (true positives) among all positive predictions, providing insight into the model’s ability to avoid false positives. Recall, on the other hand, measures the proportion of true positives detected by the model relative to all actual positive instances, reflecting its effectiveness in capturing relevant objects (Miao and Zhu, 2022).

In addition to precision and recall, Intersection-over-Union (IoU) is a crucial metric for evaluating the spatial accuracy of object detection (Wang and Song, 2021). IoU is defined as the ratio of the intersection area to the union area between a predicted bounding box and the corresponding ground truth box. A higher IoU value indicates better alignment between the predicted and actual object locations, demonstrating more precise localization.

Beyond these fundamental metrics, Average Precision (AP) and mean Average Precision (mAP) are widely used to evaluate overall detection performance (Takahashi et al., 2021). AP balances precision and recall for a specific object category, measuring the model’s ability to accurately detect objects within that category while minimizing false positives and false negatives. However, AP focuses on individual categories and does not provide a holistic view of the model’s performance across multiple classes. To address this limitation, mAP averages the AP scores across all object categories, offering a comprehensive assessment of the model’s detection capabilities (Bazame et al., 2021).

The evaluation metrics used in this study are formally defined in Equations 1–5,


(1) $$Precision=\frac{TP}{TP+FP}$$

(2) $$Recall=\frac{TP}{TP+FN}$$

(3) $$\text{IoU}=\frac{\text{Area of Overlap}}{\text{Area of Union}}$$

(4) $$AP(y,y^{*})=\frac{1}{N}\sum_{c}area(Pr)$$

(5) $$mAP=\frac{1}{n}\sum_{i=1}^{n}AP_{i}$$

In these equations: True Positive (TP) refers to instances correctly identified by the model. False Negative (FN) denotes positive instances missed by the model. False Positive (FP) represents instances incorrectly classified as positive.

A high precision score indicates a lower false positive rate, while a high recall score shows that the model successfully captures most true positives (Pratap and Kumar, 2023). IoU, as a measure of localization accuracy, ensures that the detected bounding boxes align closely with the ground truth.

This study specifically employs mAP@50, which evaluates the mean Average Precision at an IoU threshold of 0.5. This threshold strikes a balance between strict localization accuracy and the flexibility needed for robust detection in real-world scenarios. By averaging AP scores across categories, mAP@50 provides a comprehensive evaluation of the model’s ability to detect objects of varying scales and categories (Shen et al., 2022).

These metrics collectively form a robust framework for assessing the performance of target detection models. Precision, recall, and IoU measure specific aspects of detection quality, while AP and mAP offer a broader evaluation across categories. Together, they provide valuable insights into the strengths and limitations of the model, ensuring a detailed and balanced analysis of its detection capabilities.

## 4 Result and discussion

### 4.1 Ablation study

The ablation study evaluates the impact of integrating the proposed modules—C3k2_RFCBAM (N1), MDFPC (N2), and CMFPN (N3)—into the YOLOv11n+Mamba framework. **Table 2** presents the results, illustrating their influence on precision, recall, mAP@50, model size, and FPS.

**Table 2:** Performance comparison of proposed modules in ablation studies.

Model	N1: C3k2_RFCBAM	N2: MDFPC	N3: CMFPN	Precision (%)	Recall (%)	mAP@50(%)	Size(MB)	FPS
YOLOv11n	×	×	×	81.8	80.5	83.3	6.1	250
Mamba	×	×	×	75.8	78.6	80.7	12.3	61
YOLOv11n+Mamba	×	×	×	84.6	78.7	83.5	12.1	72
YOLOv11n+Mamba -N1	✓	×	×	85.7	81.2	85.1	11.4	128
YOLOv11n+Mamba -N12	✓	✓	×	85.8	84.2	86.5	15.5	167
YOLOv11n+Mamba -N123	✓	✓	✓	90.4	83.1	87.5	16.6	139

*A check mark (√) indicates the strategy module was used and a cross (×) indicates it was not used.

The baseline YOLOv11n model achieved a precision of 81.8%, recall of 80.5%, and mAP@50 of 83.3%. Despite its compact size of 6.1 MB, the model delivered the highest FPS at 250, making it efficient for real-time applications (Meribout et al., 2022). However, its limited ability to model long-range dependencies and insufficient multi-scale processing hindered its overall detection performance, particularly for small and occluded rosebuds.

Incorporating Mamba into YOLOv11n significantly enhanced detection capabilities while maintaining a lightweight architecture. The YOLOv11n+Mamba configuration improved precision to 84.6%, recall to 78.7%, and mAP@50 to 83.5%, with FPS increasing to 72. This improvement underscores Mamba’s ability to enhance long-range feature dependencies while preserving computational efficiency.

The introduction of the C3k2_RFCBAM (N1) module into the YOLOv11n+Mamba framework resulted in further performance gains. The YOLOv11n+Mamba-N1 configuration enhanced spatial feature prioritization, particularly for small-object detection, improving precision to 85.7% and recall to 81.2%, with a mAP@50 of 85.1%. This improvement came with a slight trade-off in computational efficiency, as the FPS decreased to 128 while the model size grew to 11.4 MB. These results demonstrate the ability of C3k2_RFCBAM to effectively address the challenges of occlusion and scale variability in UAV-based rose imagery.

Building on this foundation, the integration of the MDFPC (N2) module in the neck network produced the YOLOv11n+Mamba-N12 configuration. By employing dilated convolutions with varying dilation rates, the MDFPC module enhanced multi-scale feature fusion and contextual representation. This addition increased recall significantly to 84.2%, while precision improved slightly to 85.8%, resulting in a mAP@50 of 86.5%. Although the model size increased to 15.5 MB, the FPS rose to 167, highlighting the MDFPC module’s efficiency in balancing computational demands with performance improvements.

The final enhancement involved integrating the CMFPN (N3) module into the head network, creating the complete YOLOv11n+Mamba-N123 model, also referred to as ROSE-MAMBA-YOLO. This final configuration achieved the highest precision (90.4%) and mAP@50 (87.5%), while recall remained strong at 83.1%. The CMFPN module’s contextual feature integration and multi-scale fusion enabled the model to excel in detecting small and densely packed roses within complex greenhouse environments. Although this integration introduced additional computational complexity, the model size increased only modestly to 16.6 MB, and FPS remained at 139, well within the real-time threshold for UAV-based monitoring. This trade-off is justified by the substantial accuracy gains, as the improved spatial awareness and multi-scale adaptability enable more reliable detections with minimal performance loss, ensuring that ROSE-MAMBA-YOLO remains an efficient and practical solution for precision agriculture applications (Tong and Wu, 2022).

The results highlight the progressive contributions of the proposed modules. The C3k2_RFCBAM module improved small-object detection through spatial attention, the MDFPC module enhanced multi-scale feature extraction, and the CMFPN module refined contextual integration for complex scenes. Together, these modules culminate in the final ROSE-MAMBA-YOLO model, which achieves an optimal balance of accuracy, computational efficiency, and real-time performance. This makes it a robust and scalable solution for UAV-based rose detection in challenging agricultural environments.

### 4.2 Comparative experiment

To evaluate the performance of various object detection models for UAV-based rose detection, twelve models—including SSD, RT-DETR, Faster R-CNN, and multiple YOLO variants—were analyzed across precision, recall, mAP@50, model size, and FPS. All models were trained and evaluated under the same dataset, input size, and training configurations to ensure a fair comparison. The results, summarized in **Table 3**, highlight the superior performance of the proposed ROSE-MAMBA-YOLO model.

**Table 3:** Performance comparison of object detection models on the test set.

Model	Precision (%)	Recall (%)	mAP@50(%)	Size(MB)	FPS
YOLO-Worldv2	45.1	73.4	62.0	6.5	385
YOLO-Ghost-p6	63.1	65.2	65.3	4.9	333
RT-DETR	69.5	69.9	70.7	53.5	385
YOLOv6n	69.9	71.0	73.2	8.6	303
YOLOv5n	77.2	71.6	74.9	3.9	323
YOLOv10n	72.9	71.7	76.3	5.8	303
SSD	78.4	76.7	78.0	95.5	269
YOLOX-tiny	79.2	77.8	78.6	20.4	155
Faster-RCNN	80.8	74.7	79.1	113.5	44
YOLOv8n	78.2	79.4	81.2	5.6	238
YOLOv9	78.7	77.3	81.7	13.3	303
YOLOv11n	81.8	80.5	83.3	6.1	250
***Ours***	**90.4**	**83.1**	**87.5**	16.6	139

Bold values indicate the best performance across all models for each metric.

ROSE-MAMBA-YOLO achieved the highest mAP@50 of 87.5%, outperforming all tested models, including the second-ranked YOLOv11n with a mAP@50 of 83.3%. Although ROSE-MAMBA-YOLO has a lower FPS of 139 compared to YOLOv11n’s 250, it remains highly suitable for real-time UAV-based agricultural monitoring, where precision and robustness are critical (Delavarpour et al., 2021; Su et al., 2023). The model’s precision (90.4%) and recall (83.1%) underscore the effectiveness of its advanced modules—C3k2_RFCBAM, MDFPC, and CMFPN—that enhance feature extraction, multi-scale fusion, and contextual modeling.

Among the YOLO variants, YOLOv11n demonstrated strong performance with a mAP@50 of 83.3%, precision of 81.8%, and recall of 80.5%. Its compact size (6.1 MB) and high FPS of 250 make it an efficient choice for real-time applications. However, its limited ability to handle small-object detection and complex environments highlights the value of the advanced modules incorporated in ROSE-MAMBA-YOLO (Mirzaei et al., 2023). YOLOv9 and YOLOv8n also performed competitively, with mAP@50 scores of 81.7% and 81.2% and FPS values of 303 and 238, respectively. While they balance accuracy and efficiency, their lack of advanced feature fusion and contextual modeling restricts their applicability to more complex UAV-based detection tasks (Parambil et al., 2024; Wan et al., 2024).

Transformer-based models, such as RT-DETR, achieved moderate performance with a mAP@50 of 70.7% and a high FPS of 385 (Zhao et al., 2024). While their self-attention mechanisms effectively capture global dependencies, their larger model size (53.5 MB) and limited small-object detection capabilities make them less practical for real-time agricultural applications with constrained resources.

Two-stage detectors, including Faster R-CNN, showed a mAP@50 of 79.1% but were hindered by low FPS (44) and high computational complexity (113.5 MB). The reliance on a Region Proposal Network (RPN) and multi-step refinement processes adds significant computational overhead, making these detectors less suited for time-sensitive UAV tasks compared to one-stage models (Shih et al., 2019; Hou et al., 2022).

Lightweight models, such as YOLOv5n, YOLO-Ghost-p6, and YOLO-Worldv2, excelled in speed, achieving FPS values of 323, 333, and 385, respectively. However, their reduced parameter counts and simplified architectures compromised detection accuracy, with mAP@50 scores of 74.9%, 65.3%, and 62.0% (Chakrabarty et al., 2024). These results highlight the trade-off between computational efficiency and detection performance, which limits their ability to capture the intricate details of UAV-captured rose images (Huang et al., 2017; Xu et al., 2022).

**Figure 11:**
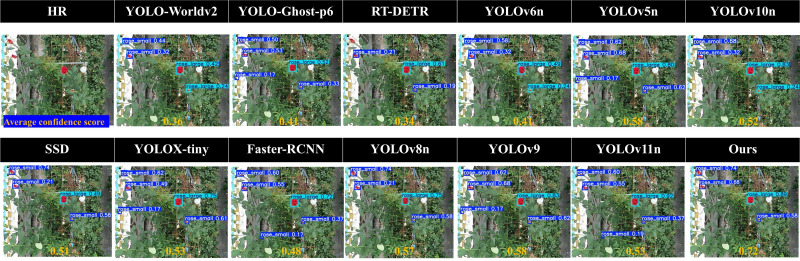
Comparison of detection results across different models.

**Figure 11** visually compares detection confidence across models. It demonstrates that ROSE-MAMBA-YOLO consistently produces higher confidence scores for rose detection, outperforming other models, especially in detecting small and partially occluded roses.

In conclusion, ROSE-MAMBA-YOLO delivers substantial improvements over existing models by addressing key challenges such as occlusions, scale variability, and complex environmental backgrounds. Despite its slightly larger size (16.6 MB) and moderate FPS, its exceptional detection accuracy and robust design make it an ideal solution for UAV-based rose monitoring (Awais et al., 2021; Telli et al., 2023). These findings validate the effectiveness of its advanced modules in tackling real-world challenges, positioning ROSE-MAMBA-YOLO as a state-of-the-art solution for precision agriculture and floriculture applications.

### 4.3 Robustness against degraded input data

**Figure 12:**
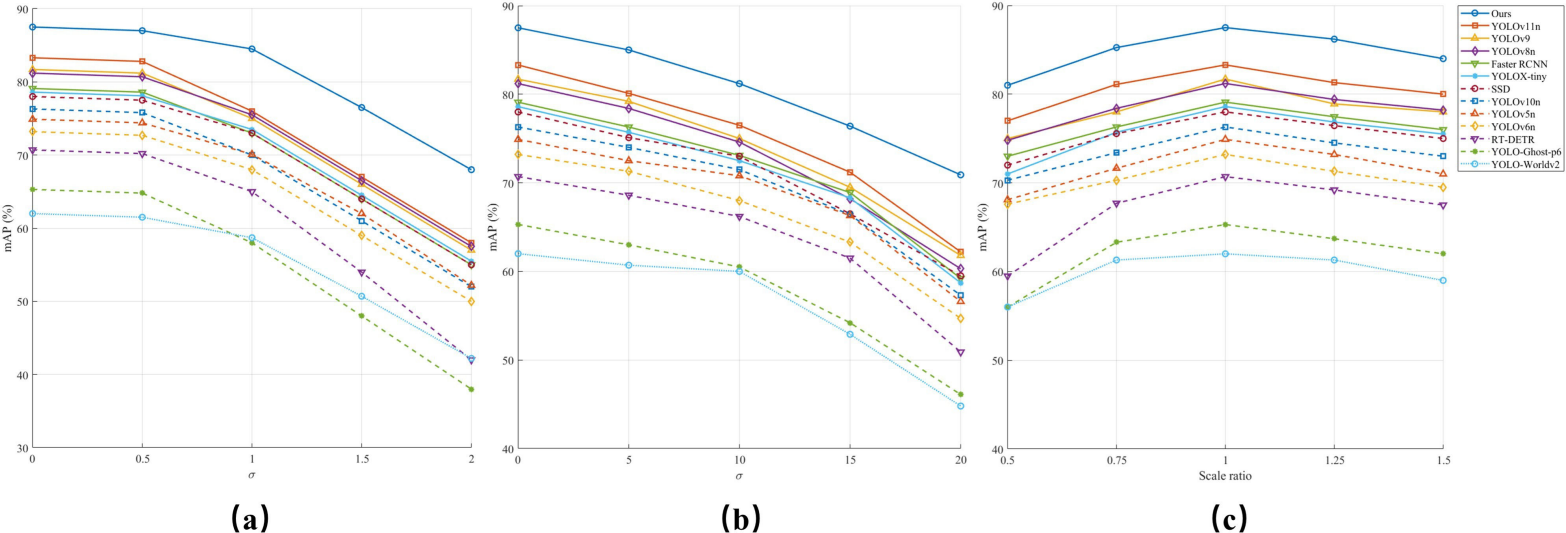
Robustness of different object detection models under degraded conditions. **(a)** Gaussian blur. **(b)** Gaussian noise. **(c)** Scaling.

In real-world applications, object detection models frequently encounter image degradation caused by factors such as blurring, noise, and scale variations (Li Y, et al., 2025). These Lichallenges can significantly affect model performance, particularly in scenarios like UAV-based monitoring and industrial inspection (Silva et al., 2024). To evaluate the robustness of Rose-Mamba-YOLO, we conducted a series of experiments simulating these degradations and compared its performance with multiple baseline models. **Figure 12** illustrate the effects of Gaussian blur, Gaussian noise, and scale variations, respectively, on the detection accuracy measured by mAP@50.

Gaussian blur is a form of distortion caused by defocusing, motion blur, or image post-processing (Flusser et al., 2015). It smooths the image by convolving it with a Gaussian kernel, reducing high-frequency details that are crucial for detecting object edges. The effect can be mathematically formulated as shown in Equation 6:


(6) $$G(x,y)=\frac{1}{2\pi \sigma^{2}}e^{-\frac{x^{2}+y^{2}}{2\sigma^{2}}}$$

As shown in **Figure 12a**, increasing the blur intensity (*σ*) leads to a consistent decline in mAP@50 across all models. However, Rose-Mamba-YOLO demonstrates superior robustness, maintaining the highest detection accuracy even under severe blurring conditions. This resilience can be attributed to the Mamba architecture’s long-range dependency modeling, which allows the model to extract essential features even when edge information is significantly degraded (Ma B, et al., 2024). Specifically, when *σ*=2, Rose-Mamba-YOLO retains over 75% of its original detection performance, whereas traditional CNN-based models like SSD and Faster RCNN suffer a drop of more than 35%. Transformer-based models, such as RT-DETR, also exhibit significant performance declines, indicating their reliance on sharp edge features for object recognition. In contrast, Rose-Mamba-YOLO’s global feature aggregation ability mitigates the loss of fine-grained details, enabling it to maintain stable performance even under extreme blurring conditions.

Gaussian noise is another common degradation factor, often arising from sensor noise or image compression artifacts (Rahimi-Ajdadi and Mollazade, 2023). The process of adding Gaussian noise is expressed in Equation 7:


(7) $$I_{noisy}(x,y)=I_{original}(x,y)+N(0,\sigma^{2})$$

where $N(0,\sigma^{2})$ represents Gaussian-distributed noise with zero mean and variance σ^2^. **Figure 12b** depicts the effect of increasing noise intensity, where all models experience performance degradation. However, Rose-Mamba-YOLO exhibits exceptional robustness, maintaining a significantly slower decline in mAP@50 compared to other models. This can be attributed to its adaptive global feature aggregation, which effectively reduces reliance on local high-frequency details that are more susceptible to noise (Liu Q, et al., 2024). In contrast, models such as YOLO-Worldv2, YOLO-Ghost-p6, and Faster RCNN struggle with distinguishing objects from the noisy background, resulting in substantial accuracy degradation.

Scale variation poses another major challenge in object detection, as objects appear at different sizes due to perspective shifts, distance changes, or variations in sensor resolution (Yang et al., 2024). Robust models must generalize across scales without requiring retraining for each scenario. **Figure 12c** presents model performance across scaling factors ranging from 0.5× to 1.5×. While most models perform optimally at the original scale, detection accuracy declines as objects shrink or enlarge. Rose-Mamba-YOLO demonstrates the strongest adaptability, maintaining stable mAP@50 among all the models, particularly at 1.5× magnification, where competing models suffer severe performance drops. This advantage stems from its Mamba-based feature extraction mechanism, which encodes multi-scale representations while preserving localization accuracy (Rahman et al., 2024). Conversely, SSD and Faster RCNN struggle significantly at 0.5×, reflecting their limitations in detecting small objects. YOLO-Worldv2 and YOLO-Ghost-p6 also exhibit sharp declines when objects deviate from the training distribution, further highlighting the importance of robust scale-invariant feature extraction (Zhang S, et al., 2023).

Beyond general robustness, Rose-Mamba-YOLO’s ability to maintain high detection accuracy under scale variations directly enhances its small-object detection capabilities. In UAV-based monitoring, objects appear smaller at higher altitudes or in wide-area views, making small-object detection inherently linked to scale variation (Heidari et al., 2023). Conventional models often fail in these scenarios due to their reliance on high-resolution details, limiting detection to later growth stages (Mulla, 2013). Rose-Mamba-YOLO’s scale-invariant detection enables the precise identification of early-stage rose buds despite their small size, occlusions, and minimal contrast against foliage. By detecting buds earlier than competing models, Rose-Mamba-YOLO extends the effective monitoring period from approximately 80% to 95% of the full flowering cycle. This improved coverage allows for more accurate tracking of growth transitions, optimizing harvesting schedules, pest control, and greenhouse climate adjustments (Balyan et al., 2024). Such advancements make Rose-Mamba-YOLO particularly valuable for large-scale commercial rose cultivation, where early and precise monitoring is critical for yield optimization and quality assurance.

These results highlight Rose-Mamba-YOLO’s potential as a state-of-the-art solution for real-world agricultural monitoring. Its robust feature extraction, small-object detection efficiency, and scalability make it well-suited for large-scale greenhouse cultivation, UAV-based precision agriculture, and automated crop monitoring. The integration of Mamba’s state-space modeling within YOLO’s efficient detection framework ensures reliable performance under diverse environmental conditions, paving the way for more advanced and data-driven agricultural applications.

## 5 Conclusions

This study introduces ROSE-MAMBA-YOLO, a detection model specifically designed to address the challenges of UAV-based rose detection in greenhouse environments. By integrating Mamba-inspired state-space modules into the YOLOv11 framework, the model achieves notable improvements in feature extraction, multi-scale fusion, and contextual understanding, enabling accurate detection of roses across different growth stages. These advancements effectively address key issues such as occlusions, scale variability, and complex environmental conditions.

Experimental results highlight ROSE-MAMBA-YOLO’s superior performance, achieving a mAP@50 of 87.5% with precision and recall values of 90.4% and 83.1%. Its lightweight design (16.6 MB) and computational efficiency establish it as a scalable solution for UAV-based agricultural applications. The inclusion of modules such as C3k2_RFCBAM, MDFPC, and CMFPN enhances its capability to detect small objects and navigate challenging scenarios, ensuring reliability in real-world settings. Robustness evaluation under Gaussian blur, Gaussian noise, and scale variations demonstrated its resilience compared to CNN-based and Transformer-based models. Despite increased blur intensity, ROSE-MAMBA-YOLO retained essential edge details, mitigating high-frequency feature loss. Extensive testing confirmed its adaptability to diverse datasets and robustness against degraded input data, demonstrating its potential for broader agricultural monitoring tasks.

This research provides a practical and efficient solution for UAV-based rose monitoring, paving the way for intelligent and data-driven precision agriculture. While this study adopts a binary classification scheme for simplicity and real-time deployment, future work will explore extending the model to support finer-grained growth stage distinctions and additional flower species to better meet practical agricultural needs. ROSE-MAMBA-YOLO’s integration into UAVs, agricultural robots, or mobile systems promises to revolutionize crop monitoring and advance the development of precision floriculture practices.

## Data Availability

Publicly available datasets were analyzed in this study. This data can be found here: https://github.com/dahlian00/RoseBlooming-Dataset.
